# Toxic Effects and Tumor Promotion Activity of Marine Phytoplankton Toxins: A Review

**DOI:** 10.3390/toxins14060397

**Published:** 2022-06-08

**Authors:** Biswajita Pradhan, Hansol Kim, Sofia Abassi, Jang-Seu Ki

**Affiliations:** Department of Biotechnology, Sangmyung University, Seoul 03016, Korea; pradhan.biswajita2014@gmail.com (B.P.); 201934001@sangmyung.kr (H.K.); 201634026@sangmyung.kr (S.A.)

**Keywords:** phytoplankton, toxin, toxic effects, clinical symptoms, reactive oxygen species (ROS)

## Abstract

Phytoplankton are photosynthetic microorganisms in aquatic environments that produce many bioactive substances. However, some of them are toxic to aquatic organisms via filter-feeding and are even poisonous to humans through the food chain. Human poisoning from these substances and their serious long-term consequences have resulted in several health threats, including cancer, skin disorders, and other diseases, which have been frequently documented. Seafood poisoning disorders triggered by phytoplankton toxins include paralytic shellfish poisoning (PSP), neurotoxic shellfish poisoning (NSP), amnesic shellfish poisoning (ASP), diarrheic shellfish poisoning (DSP), ciguatera fish poisoning (CFP), and azaspiracid shellfish poisoning (AZP). Accordingly, identifying harmful shellfish poisoning and toxin-producing species and their detrimental effects is urgently required. Although the harmful effects of these toxins are well documented, their possible modes of action are insufficiently understood in terms of clinical symptoms. In this review, we summarize the current state of knowledge regarding phytoplankton toxins and their detrimental consequences, including tumor-promoting activity. The structure, source, and clinical symptoms caused by these toxins, as well as their molecular mechanisms of action on voltage-gated ion channels, are briefly discussed. Moreover, the possible stress-associated reactive oxygen species (ROS)-related modes of action are summarized. Finally, we describe the toxic effects of phytoplankton toxins and discuss future research in the field of stress-associated ROS-related toxicity. Moreover, these toxins can also be used in different pharmacological prospects and can be established as a potent pharmacophore in the near future.

## 1. Introduction

Phytoplankton, typically found in aquatic systems, are microscopic, unicellular organisms that exist solitarily or in chains and are photosynthetic. The activities of freshwater and marine water Cyanobacteria, diatoms, and dinoflagellates may account for almost half of the global CO_2_ fixation [1,2,3,4,5,6]. However, some phytoplankton can multiply rapidly to form harmful algal blooms (HABs), and some even produce toxins that harm marine life and humans.

Phytoplankton are the chief contributor of toxins [7,8,9] and they are responsible for a variety of human ailments associated with seafood consumption [10]. They have been linked to episodic deaths in humans (1.5%) and other organisms, such as marine birds, mammals, and organisms dependent on the marine food web [11]. Phytoplankton cause blooms and are the main source of toxins in response to environmental conditions. Hazardous diatoms such as *Pseudo-nitzschia* are frequently found and are the chief contributors to wreaking havoc on the environment, aquatic organisms, and humans [12]. To date, only 2% (0–80 species) of over 3400–4000 phytoplankton taxa are established [10,13]. Cyanobacteria, diatoms, and dinoflagellates are the chief contributors to phytoplankton toxins that are harmful to humans and other aquatic organisms [11]. Filter-feeding shellfish, herbivorous fish, and zooplankton consume phytoplankton and serve as mediators for humans either directly (shellfish) or indirectly (zooplankton) through the food web. Phytoplankton toxins are responsible for several seafood poisoning disorders, including paralytic shellfish poisoning (PSP), neurotoxic shellfish poisoning (NSP), amnesic shellfish poisoning (ASP), diarrheic shellfish poisoning (DSP), and ciguatera fish poisoning (CFP) [11]. The majority of the neurotoxins are heat-stable, and cooking temperature is not enough to completely inactivate these toxins. In addition to foodborne poisoning, toxins from some dinoflagellates can be aerosolized (brevetoxins) or volatilized (a putative Pfiesteria toxin) and are harmful to the human respiratory system [11]. While certain phytoplankton toxins are toxic, others may pose various threats.

According to several epidemiological and experimental studies, chronic exposure to phytoplankton toxins in humans has been linked to carcinogenesis, particularly in the skin, lungs, nasopharynx, pancreas, kidneys, breast, prostate, urinary bladder, and hematological systems [14]. Phytoplankton toxins can induce several clinical symptoms. These include abdominal pain, vomiting, diarrhea, severe headaches, confusion, agitation, somnolence (sleepiness), memory loss, coma, ataxia (incoordination), excessive scratching, tremors, heart, seizures, spells of significant lethargy, inappetence, central blindness, vomiting, blepharospasm, muscular twitching, aberrant behavioral difficulties, convulsions, and mortality [15]. Moreover, it is critical to investigate their chemical origins and environmental effects in the context of overall health. Phytotoxins have attracted scientific interest because of their increasing impact on ecosystems, animals, and humans. Understanding the chemical and physical features of these toxins, their presence in marine waters, production management, and their fate in ecosystems is important for assessing the specific effects of these toxins and their possible mechanisms of action, which are urgently needed. Lung, skin, breast, prostate, pancreas, urinary bladder, and nasopharyngeal cancers have all been linked to exposure to phytoplankton toxins. In mammals, microcystins and nodularin are unique liver poisons [16], and acute exposure to both results in hepatic bleeding and failure [16,17]. The okadaic acid pathway induces cancers in the skin, liver, and glandular stomach of mice and rats [17,18,19].

In this review, we focus on the human clinical symptoms produced by phytoplankton toxins and their possible mechanisms of toxicity. In addition, while the impacts of phytoplankton toxicity on human health have yet to be completely explored, the probable mechanisms of toxicity related to reactive oxygen species (ROS) and their tumor promotion activity are underlined. The neurotoxic effects of phytoplankton toxins are also briefly discussed.

## 2. Marine Phytoplankton: The Most Important Source of Toxins

**Figure 1:**
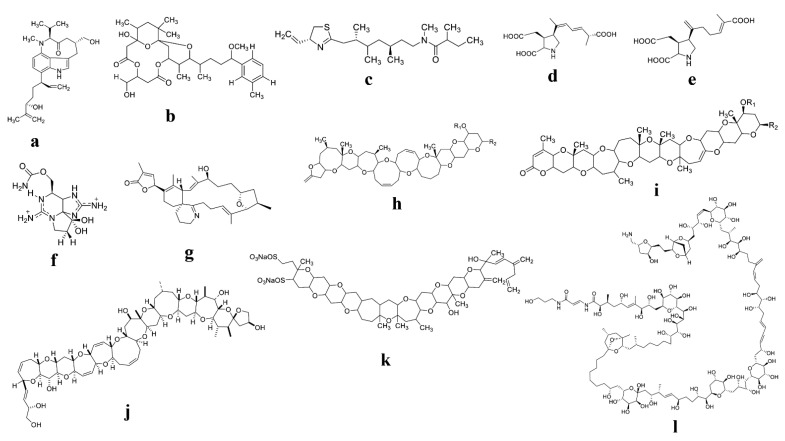
Molecular structures of different hazardous toxins derived from phytoplankton; (**a**) Lyngbyatoxin; (**b**) Oscillatoxins; (**c**) Kalkitoxin; (**d**) Domoic acid; (**e**) Iso-domoic acid; (**f**) Saxitoxin (STX); (**g**) spirolide C; (**h**) Brevetoxin type-A; (**i**) Brevetoxin type-B; (**j**) Ciguatoxin; (**k**) Palytoxin; (**l**) Yessotoxin.

One of the most important components of marine ecosystems is phytoplankton [1], and because of their photosynthetic activities, they play an important role as the principal source of biomass and organic molecules in oceans [20,21]. In contrast, many phytoplankton species produce secondary bioactive metabolites, including poisonous toxins [22,23,24,25]. The most hazardous molecular structures of certain toxins derived from marine phytoplankton are displayed in Figure 1. However, the evolutionary and functional relevance of these toxins remain unknown. They can be discharged into the environment and exert allelochemical effects to combat rivals or grazers [20]. Phytoplankton toxins are mostly neurotoxins with various chemical structures, ranging from comparatively simple alkaloids and amino acids to polyketides, a family of extremely diverse compound structures and toxic effects. In addition, maitotoxin and palytoxin are toxins generated by the dinoflagellates *Ostreopsis siamensis* and *Gambierdiscus toxicus* [11]. Toxins accumulate in filter-feeding fish and shellfish, causing PSP, ASP, DSP, CFP, and azaspiracid shellfish poisoning (AZP). In addition to human ailments induced by consuming contaminated seafood, certain marine toxins, such as tetrodotoxin, have the potential to be used in bioterrorism [26]. Saxitoxin and its analogs are the most toxic among marine neurotoxins [27]. In addition, they also produce polypeptides, which are neurotoxins that target the sodium channels.

## 3. Shellfish Poisoning Toxins: The Most Hazardous Impact to Human Health

Epidemiological studies have reported human disorders caused by marine dinoflagellate toxins, and most cases are well-documented [28]. PSP, DSP, CFP, NSP, and AZP are disorders caused by shellfish poisoning. Symptoms usually appear due to eating contaminated seafood or exposure to toxins directly or via HABs [29]. The most frequent marine toxin is ciguatera poisoning. Ciguatera toxin can be detected in the plasma, serum, and urine of patients. Moreover, fish populations can be affected because the larvae have a lower chance of surviving ciguatera toxicity [30]. Saxitoxin (STX) pufferfish poisoning (PFP) causes a similar disease; however, bioaccumulation occurs in pufferfish rather than shellfish. The active toxin was identified as STX and two of its variants, with Pyrodinium bahamense being the main producer. PFP is usually linked to tetrodotoxin, whereas PSP is named after STX pufferfish poisoning (SPFP), which causes food poisoning [31].

PSP is a life-threatening syndrome caused by shellfish poisoning that has been documented worldwide [32]. Filter-feeding mollusks and crustaceans swallow harmful cells in the event of PSP, which concentrates the toxin inside the animal’s organs and tissues. The first PSP outbreak was reported in 1927 near San Francisco, California when Alexandrium catenella caused 106 cases and six deaths [33]. Since then, members of the genera Gymnodinium, Pyrodinium, and Alexandrium have been identified as important causes of PSP, while the majority of PSP outbreaks are caused by consuming contaminated shellfish. Numbness and paresthesia, first around the mouth and lips and then the neck and face, muscle weakness, a feeling of lightness and floating, lethargy, motor incoordination, ataxia, incoherence, and a steady decrease in ventilator efficiency are all symptoms of PSP. These complications eventually lead to respiratory paralysis and death in patients with severe intoxication [34]. Every year, nearly 2000 cases of human PSP are reported worldwide, with a 15% mortality rate [35]. PSP has also been linked to the death of marine whales, birds, and monk seals [36].

Certain seafood species have evolved to survive in high quantities of algal toxins. Softshell clams from red tide-affected areas are more resistant to PSP poisoning, and toxins accumulate at higher rates in them than in sensitive clams from unaffected areas [37]. Humans may be at risk due to this because after accumulation, toxins go through a number of biotransformations in the hosts, and the impact of the modified toxins on humans has not yet been thoroughly investigated [15]. In addition to well-known toxins, dinoflagellates produce some of the largest and most complex toxins ever discovered that modulates tumorigenic and neurotoxic actions, and are produced by synthase genes [38]. Dinoflagellate toxins are gaining popularity because of their wide spectrum of toxic effects [39].

STX and its analogs were discovered in shellfish, where they were initially concentrated by marine dinoflagellates and have been linked to human deaths [40]. However, cyanobacteria, such as Lyngbya wollei, Cylindrospermopsis raciborskii, Anabaena circinalis, and Aphanizomenon flos-aquae, have all been shown to produce STX. The NH-1 and NH-5 strains of Aphanizomenon flos-aquae from North America primarily contain neosaxitoxin (NEO) and less STX, as well as a few unidentified neurotoxins. Although some cyanobacterial toxins, such as nodularin, have been found in brackish water and neurotoxic factors have been found in marine environments, the presence of the neurotoxin β-methylamino-L-alanine (BMAA) in saline habitats could be due to its presence inside the Microcystis bloom [41]. BMAA and its isomers and marine toxins such as brevetoxin may be produced by diatoms and dinoflagellates in oceans. Risk evaluation and monitoring are essential because of the possibility of numerous co-occurrences and co-exposures to brevetoxins, microcystins, and BMAA. Although BMAA and its isomers have been discovered in marine blooms involving diatoms and dinoflagellates, more research is needed to fully understand the presence of this cyanobacterial toxin in these algal species [42].

However, no vaccines are available to guard against phytoplankton toxicity. For example, an experiment was conducted on the tetrodotoxin (TTX) vaccination of mice. To make false antigens TTX–TT and TTX–TTH, the vaccine used Tachypleus tridentatus hemocyanin (TTH) and tetanus toxoid (TT) as carrier proteins. The TTH–TTX vaccine protected mice against orally administered TTX better than the TTX–TT vaccine [43,44]. Initially, it was shown that new experimental vaccines could protect animals from repeated ingestion of marine phytoplankton toxins. Not only do marine phytoplankton toxins cause seafood poisoning, but they also induce skin, liver, hepatic, and gastrointestinal tumor promotion activity. Additionally, the toxins cause allergic reactions, irritants, headaches, and several other diseases. Furthermore, phytotoxins cause stress-associated ROS-related diseases and cancers, which pose a serious threat and may lead to death.

### 3.1. Marine Cyanobacterial Toxins Association with Clinical Symptoms

Marine cyanobacteria are key sources of bioactive and harmful toxins (Table 1). Toxin-contaminated water by several species of cyanobacteria often causes acute and sometimes serious diseases as well as lethal illnesses in humans and other organisms such as cattle, birds, pets, wildlife, and fish. Several countries have reported losses due to toxins impacting organisms and the marine tourism health sector. However, the expanding pool of toxins produced by marine cyanophytes provides a unique supply of bioactive compounds for toxicological research [45].

Cyanobacterial toxins in the ocean can cause contact dermatitis and gastrointestinal diseases in humans, mainly in swimmers, and Lyngbya majuscula is one of the most common culprits [58]. The brominated alkaloid aplysiatoxin and its derivatives elicit an inflammatory response when they are in contact with the skin, and they are responsible for serious food poisoning. Aplysiatoxin is produced by *Schizothrix calcicola* and *Oscillatoria nigroviridis*, which cause a burning feeling in the throat and mouth, paresthesia, abdominal pain, vomiting, diarrhea, convulsions, and low blood pressure in humans. They are also potent tumor promoters [51]. Aplysiatoxin analogs significantly increased phospho-PKCδ expression and selectively blocked the potassium channel Kv1.5 [52]. Lyngbyatoxin A is a dermatotoxic alkaloid generated by *L. majuscula* and it has a similar structure to teleocidin A-1, a severe skin irritant and tumor promoter with similar toxicity to aplysiatoxin. Because of their lower affinity for phorbol ester receptors, lyngbyatoxin B and C, which are also found in *L. majuscula*, are thought to be weaker tumor promoters than lyngbyatoxin-A [49]. In humans, lyngbyatoxin-A causes dermatitis and oral and gastrointestinal inflammation [59]. Oscillatoxin produced by *Schizothrix calcicola* and *Oscillatoria nigroviridis* causes contact irritation [48]. Contact with lipopolysaccharides (LPSs), which are produced by cyanobacteria, elicits allergic, inflammatory, and pyrogenic reactions in humans and other animals [60]. Moreover, it acts as a powerful tumor promoter and protein kinase C activator in humans [50]. In mammals, LPS causes fever and is involved in septic shock syndrome [61]. Although the actual mechanism of tumor promotion is still unclear, cyanobacterial toxins cause an increase in oxidative stress, leading to an increase in ROS, which can damage DNA and has been linked to phytotoxin-induced liver cancer [62,63,64].

Despite being widespread, mainly due to physical contact, marine cyanobacterial neurotoxins have also been found in edible fish, posing a risk to humans [65]. Several neurotoxic chemicals have been identified in marine cyanobacteria. Kalkitoxin, most recently found in *L. majuscula* and *Trichodesmium* spp., causes rapid neurotoxicity and neuronal necrosis in rat cerebellar neurons through an N-methyl-D-aspartate receptor pathway and is related to STX, which are a group of carbamate alkaloids with potent sodium-channel blockage capabilities that have been linked to human death [53]. Moreover, it is ichthyotoxic to goldfish such as *Carassius auratus* and toxic to crustaceans such as *Artemia salina* [66]. Antillatoxin from *L. majuscula* displayed strong ichthyotoxicity and neurotoxicity (EC_50_ = 20.1 ± 6.4 nM) [54]. *Nodularia spumigena* primarily produces nodularin in brackish waters. It has a similar structure and mechanism of toxicity to microcystins [64,67]. Furthermore, nodularin from naturally occurring phytoplankton samples, such as *N. spumigena* from the Baltic Sea, modulates the toxicity of human and rat hepatocytes by inhibiting the activity of protein phosphatase 1 and 2A [64]. Individuals affected by this toxin experience symptoms such as renal lesions, diarrhea, vomiting, piloerection, weakness, and pallor [46,47]. The marine cyanobacterial toxins and their toxic targets and associated clinical symptoms are shown in Table 1.

### 3.2. Marine Diatoms Toxins and Their Toxic Effects and Clinical Symptoms

Domoic acid (DA) is a secondary metabolite with a structure similar to that of kainic acid and amino acids, such as aspartic and glutamic acid. Many diatom species, such as the genus *Pseudo-nitzschia*, produce domoic acid, and its toxic targets and related clinical symptoms are displayed in Table 1. It is a non-protein amino acid that is crystalline, water-soluble and has a molecular weight of 311 Da [68]. In addition to *Pseudo-nitzschia*, a second diatom genus, Amphora, has been identified to produce DA [69]. Moreover, Antarctic diatom species have been identified as DA producers [70]. The genus *Pseudo-nitzschia* is a marine planktonic diatom with 30 species, 12 of which are well-known DA producers. *Pseudo-nitzschia* spp. may produce more toxins due to various changes in the concentrations of iron, silicon, copper, phosphorus, and nitrogen, as well as higher carbon dioxide concentrations. In addition, stress conditions can enhance the formation of DA in diatoms. Toxigenic diatoms cause poisoning in both humans and animals on a regular basis. Furthermore, domoic acid has poisoned fish-eating birds, marine mammals, and humans [71]. DA can permanently harm the nervous system and is accountable for ASP, which has been demonstrated to cause oxidative stress, mitochondrial damage, and death. In many instances, ASP has been observed in seagulls, marine animals, sea lions, and fish, resulting in various symptoms [72]. Common symptoms in patients affected by this toxin include abdominal pain, vomiting, diarrhea, severe headaches, confusion, agitation, somnolence (sleepiness), memory loss, coma, ataxia (incoordination), excessive scratching, tremors, heart difficulties, convulsions, and death [71]. Furthermore, DA is noxious to neuronal cells, as demonstrated by in vitro studies [55]. DA toxicity manifests in a variety of clinical symptoms, including brain pathology, tissue/cell injury, and memory loss [73,74]. Clinical symptoms and brain lesions observed in animal toxicology studies are frequently similar to those observed in naturally exposed species such as sea lions and humans. These clinical symptoms include seizures, spells of significant lethargy and inappetence, central blindness, vomiting, blepharospasm, muscular twitching, and aberrant behavior [75].

### 3.3. Marine Dinoflagellates Toxins and Their Toxic Effects and Clinical Symptoms

Dinoflagellate species produce diverse toxins, the majority of which are neurotoxic, killing large numbers of fish, birds, and marine mammals, with some also causing human casualties. In addition, these toxins exhibit cancer-causing properties and are associated with other stress-related diseases. *Alexandrium*, *Gymnodinium*, and *Pyrodinium* are toxin-producing dinoflagellates [11]. Their toxins, disease-causing clinical symptoms, and potential targets, including molecular mechanisms, are summarized in (Table 2).

Marine dinoflagellates produce STX. This toxin is an alkaloid with a molecular weight of 299 Da and is generally known as PSP. The toxicity of STX derivatives varies depending on their types; among these, the most dangerous compounds are STX, NEO, and gonyautoxins (GTX1-4). STX binds to the sodium and calcium channels in nerve axon membranes and prevents these ions from passing over the cell membrane, thereby inhibiting nerve impulse transmission in nerves extending to the heart cells [56,57]. STX poisoning can induce symptoms such as tingling and numbness around the lips, neuromuscular paralysis, and death due to respiratory failure. It also induces a cardio-depressive effect [57]. STX is the most dangerous toxin, and its neurotoxic effects have been well studied. The LD_50_ is 3–10 µg/kg body weight in mice, while the LD_50_ after oral administration is 263 µg/kg body weight. In humans, the lethal oral dose ranges from 1 to 4 mg, depending on the sex and physiological state of the patient. It is quickly absorbed and eliminated via the urine after passing through the intestinal tract [11].

The symptoms of STX toxicity include tickling sensations in the mouth, lips, and tongue, numbness in the extremities, breathing difficulties, gastrointestinal problems, and a sense of detachment followed by complete paralysis [90]. STX causes various neurological symptoms that lead to respiratory arrest, cardiovascular shock, and death in cases of acute intoxication [90]. The toxins bind with high affinity (Kd~2 nM) to receptor site 1 on the outside surface of the membrane and very close to the external orifice of the voltage-dependent sodium channel, stopping sodium ions from passing over the nerve cell membranes and thus interfering with signal transmission along the nerves. The resulting widespread obstruction prevents impulse generation in the peripheral neurons and skeletal muscles. STX also directly affects skeletal muscle by inhibiting the muscle action potential without depolarizing cells, effectively stopping peripheral nerve transmission, but without curare-like activity at the neuromuscular junction, leading to neural dysfunction [37]. STX toxicity related to neurotoxicity has been well studied; hence, studies should focus on tumor promotion and other stress-associated toxicities, which is an emergent area of study.

*Karenia brevis* produces brevetoxin, which causes mild gastroenteritis with neurologic signs and death in birds, large fish, and marine animals. Other symptoms include nausea, tingling, numbness in the perioral area, loss of motor function, and acute muscular pain [91]. Brevetoxins have been extensively investigated and are thought to be depolarizing chemicals that open voltage-gated sodium ion channels in the cell membranes, allowing unregulated Na^+^ influx into the cell. The toxins inhibit channel inactivation by blocking the sodium channel and preventing sodium ions from flowing over nerve cell membranes [85]. Moreover, no studies have been conducted on tumor promotion activity and stress-associated ROS-related toxicity.

Ciguatoxin (CTX) is a fat-soluble toxin generated by specific benthic strains of *Gambierdiscus toxicus*. It is one of a series of marine polycyclic ether physiologically active toxins linked to ciguatera fish poisoning outbreaks [92]. It builds up in the food chain, causing neurological, gastrointestinal, and cardiovascular problems in humans [82,83,84]. CTX and its 20 counterparts have been discovered in the Caribbean and Indian Ocean waters with small molecular variations and toxicity [83,84]. This causes a decrease in the nerve conduction rate and amplitude in human nerves, which is steady with aberrant and prolonged Na^+^ channel opening in neuronal membranes [93,94]. *Gambierdiscus toxicus* produces CTX and maitotoxin, which are lethal in mice at 0.15 and 0.45 μg/kg body weight, respectively. The toxic oral dose in adult humans is 0.1 μg. CTX causes an increase in intracellular calcium, which acts as a second messenger in the cell and disrupts critical ion-exchange mechanisms, resulting in fluid discharge and diarrhea [83]. Therefore, studies should focus on gastrointestinal and cardiovascular problems and other stress-related toxicity-related tumor promotion activities.

*Protoperidinium crassipes* produces the azaspiracid (AZA) toxin, which poses several threats to human health. Symptoms of AZA intoxication includes severe diarrhea, vomiting, nausea, and stomach cramps. Neurotoxic symptoms have also been noted [81]. Repeated injections of AZA in mice can result in the growth of lung tumors. It also induces necrosis in the *Lamina propria* of the small intestine, as well as in lymphoid tissues such as the spleen, thymus, and Peyer’s patches [95]. The mechanism of action of AZA remains unknown. More studies are urgently required to understand the actual mechanism of lung tumor promotion. However, the limited availability of pure AZA has hampered research in this area.

*Gonyaulax spinifera*, *Lingulodinium polyedrum*, and *Protoceratium reticulatum* generate yessotoxins (YTXs) [96]. YTX causes motor discoordination in mice before death [86]. Moreover, the toxin is a powerful neurotoxin. However, the primary site of action and mechanism of action remain to be elucidated [97]. Additional studies are necessary to determine the actual mechanisms and other related carcinogenic activities.

Palytoxin (PLTX)-like compounds formed by dinoflagellates of the genus *Ostreopsis*, such as *Ostreopsis mascarenensis*, *O. siamensis*, *O. lenticularis*, *O. fattorussoi*, and *O. ovata*, are usually known as ostreocin and are quite toxic to mammals [98]. These chemicals inhibit the ATPase Na^+^/K^+^ pump and block the electrochemical gradient created across the cell membrane, thereby affecting cellular activity [87,88]. PTX is a powerful toxin, with LD_50_ ranging from 0.025 µg/kg in rabbits and dogs, 0.45 µg/kg in mice, and 0.9 µg/kg in guinea pigs, and also affecting rats and monkeys. Fever, ataxia, inactivity, drowsiness, and limb weakness are the symptoms of PLTX toxicity, which may lead to death [99]. Further studies are required to understand the mechanism of action.

Spirolides (SPX) are biologically active toxins generated by *Alexandrium ostenfeldii*, *Alexandrium peruvianum*, and *Karenia selliformis*, with 16 isoforms currently identified [100,101,102,103,104]. SPX toxins have been shown to significantly affect muscarinic and nicotinic acetylcholine receptors and damage neurons and astrocytes, all of which severely affect the central nervous system [89]. Further research is required to understand the precise mechanism of toxicity.

Dinoflagellates, such as *Dinophysis* spp. and *Prorocentrum lima*, produce okadaic acid and dinophysistoxins. In the cytoplasm of mammalian cells, lipophilic okadaic acid inhibits protein phosphorylase phosphatase-1 and -2A, which dephosphorylate serine and threonine. Symptoms caused by the toxicity induced by this toxin include incapacitating diarrhea, nausea, vomiting, and abdominal pain [76]. Moreover, they also bind to Ser/Thr protein phosphatases and exert toxicity [80]. Further research is required to understand the precise mechanism underlying its toxicity.

## 4. Tumor Promotion Activity by Marine Phytoplankton Toxins

**Figure 2:**
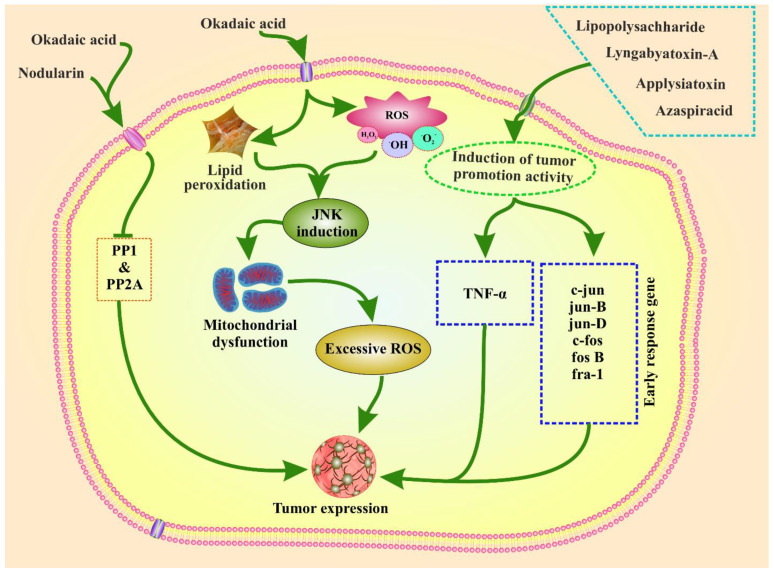
Toxicity pathway and tumor-promotion activity of phytoplankton toxins. Phytoplankton toxins such as nodularin and okadaic acid (OA) bind the protein phosphatase (PP), which triggers the inactivation of PP, further leading to tumor formation. OA causes lipid peroxidation, and ROS generation has also been examined in the context of the JNK pathway, which causes mitochondrial dysfunction and leads to excessive ROS production and tumor formation. Moreover, lipopolysaccharide, lyngbyatoxin-A, aplysiatoxin, and azaspiracid can cause tumor formation by triggering the production of tumor necrosis factor-α (TNF-α) and inducing early response genes.

Phytoplankton toxins can promote tumor growth and ROS-induced toxicity in animals. The mechanism of tumor-promoting activity of the toxins is shown in Figure 2. Phytoplankton toxins trigger ROS production. ROS generation by microcystins has been examined in the context of the c-Jun N-terminal protein kinase (JNK) pathway. JNK activation causes mitochondrial failure, which leads to hepatocyte apoptosis and liver injury in rats, and has been demonstrated to occur in the presence of microcystins and okadaic acid [78,105,106]. Aplysiatoxin, nodularin, lyngbyatoxin A, LPS, AZA, and okadaic acid derived from phytoplankton have the potential to promote tumor growth. Similar to microcystins, nodularin also promotes tumors in the liver, skin, and glandular stomach of mice, which also inhibits PP1 and PP2A and triggers tumor initiation [17,18,19]. In animal studies, nodularin can promote the production of tumor necrosis factor-α (TNF-α) and induce early response genes, namely, jun B, jun D, c-fos, c-jun, fos B, and fra-1 in the rat liver. This induction leads to tumor expression. Moreover, TNF-α has been proposed as an endogenous tumor promoter involved in human cancer development and it can trigger tumor promotion in humans [18,19].

## 5. Possible Role of Marine Phytoplankton Toxins in Oxidative Stress Related ROS Toxicity

Oxidative stress occurs when there is a mismatch between the systemic manifestation of ROS and the biological system’s ability to quickly detoxify reactive intermediates or be unable to repair the damage. ROS include superoxide radicals (^•^O_2_^−^), hydrogen peroxide (H_2_O_2_), and hydroxyl radicals (^•^OH), which are generated as metabolic byproducts in biological systems [107,108,109]. The generation of ROS and its mechanism of action are poorly understood [110] and have been poorly studied [111]. Toxins can interfere with enzymes, leading to the failure of antioxidant systems. This may lead to the failure of energy generation and oxidative stress protection. Exposure to these agents has powerful cumulative effects on humans, including decreased sperm count, aging, and other health conditions [112]. Chronic absorption of these toxins in the gastrointestinal tract is one of the most hazardous effects of multidirectional toxicity [90].

Phytoplankton toxins, such as microcystin, have a strong binding affinity with cysteine, glutathione, GSH, and reduced glutathione GS-SG, disrupting their normal functions [105,106]. Biologically essential macromolecules, such as proteins, lipids, DNA, and cellular membrane phospholipids, are oxidized and damaged by phytotoxin-induced oxidative stress. Moreover, microcystin inhibits oxidative phosphorylation and ATP generation by reducing the potential of the mitochondrial membranes. Microcystin also damages DNA and inhibits several DNA repair enzymes. Moreover, phytoplankton toxins such as microcystin, nodularin, and okadaic acid alter cell signaling pathways, affect gene expression, and promote cancer [79,106,107].

**Figure 3:**
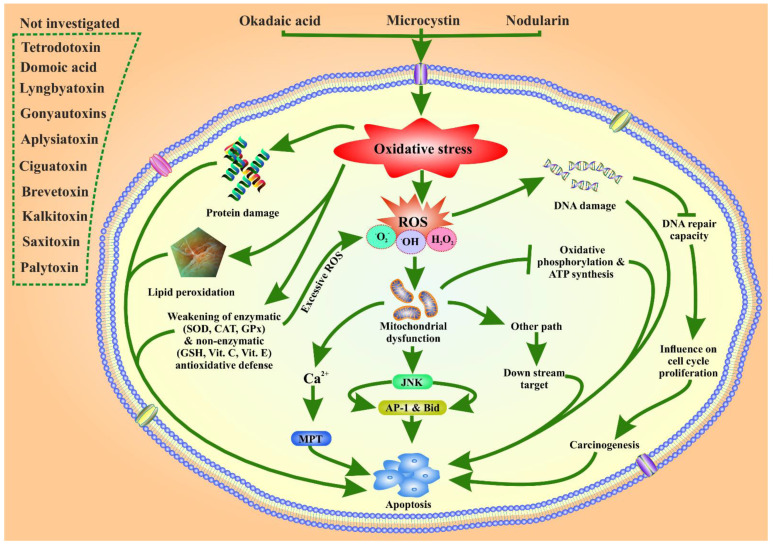
Okadaic acid (OA), microcystin and nodularin are involved in oxidative stress and the generation of reactive oxygen species (ROS), resulting in several toxicities to organisms. The toxins induce oxidative stress, damage macromolecules such as proteins, lipids, and DNA, and inhibit protein folding activity. It also decreases the activity of DNA repair enzymes that influence cell cycle proliferation and trigger carcinogenesis. OA and nodularin weaken the enzymatic and non-enzymatic antioxidant enzymes, triggering apoptosis and excessive ROS production. ROS generation has also been examined in the context of the JNK pathway leading to apoptosis via activating downstream transcription factors AP-1 and Bid. ROS causes mitochondrial dysfunction, induces the mitochondrial permeability transition (MPT) pathway, a Ca^2+^ dependent pathway, and triggers apoptosis. Mitochondrial dysfunction inhibits oxidative phosphorylation and ATP synthesis, which finally causes apoptosis. Eventually, ROS enters other pathways and triggers apoptosis.

Oxidative damage causes an increase in ROS production and impedes the electron flow through complex III, which blocks the mitochondrial electron transfer chain. Excessive ROS generation can result in macromolecule oxidation, mtDNA mutations, depolarization of the mitochondrial membrane, and apoptosis. Phytoplankton toxins trigger ROS production, which is generally counteracted by enzymatic (CAT, GPx, and SOD) and non-enzymatic (GSH, vitamin C, and vitamin E) antioxidant barriers. This has been confirmed in microcystin-LR and nodularin phytoplankton toxins [62,63,64]. Okadaic acid elicits both extracellular and intracellular ROS production in human and rat neutrophils at a minimum concentration of 10 nM [78]. Okadaic acid inhibits protein phosphatases, causes oxidative damage, and disrupts a variety of biological activities, including the cell cycle, gene expression, and DNA repair pathways [77]. Moreover, it inhibits the activity of protein phosphatases 1 and 2A, which induce carcinogenesis [64]. Oxidative stress triggers the JNK pathway and activates downstream transcription factors AP-1 and BH3-interacting domain death agonist (Bid) in the context of microcystin-LR-induced liver damage in mice via ROS. Excessive ROS induced by nodularin and okadaic acid activates the mitochondrial permeability transition (MPT) pathway by increasing Ca^2+^, which leads to apoptosis [112]. Phytoplankton toxins trigger apoptosis and necrosis [62,63,64], but the precise mechanism of ROS-associated toxicity by phytoplankton toxins remains unknown. The possible mechanisms postulated from the existing literature are displayed and summarized in Figure 3. Among these toxins, okadaic acid and nodularin involvement in stress-associated ROS-related toxicity have been well studied [77,111,113]. Studies on stress-associated ROS-related toxicity of other toxins, such as STX, kalkitoxin, brevetoxin, aplysiatoxin, ciguatoxin, domoic acid, palytoxin, gonyautoxins, and lyngbyatoxin, have not yet been investigated. Therefore, further research is urgently needed to generate a comprehensive conclusion about the toxic effects of phytoplankton toxins to generate oxidative stress-related ROS toxicity, and its tumor-promoting activity.

## 6. Phytoplankton Toxin and Their Disease Preventing Activities

Searching of natural compounds in disease various prevention is most promising [114,115,116,117,118,119,120,121]. In this regard, phytoplankton are gaining much attention [122]. Although phytoplankton toxins are poisonous to organisms, they are increasingly being investigated as a potential therapeutic for diseases including cancer, Alzheimer’s disease, AIDS, diabetes, and others [123]. Oscillatoxin and its analogs from the cyanobacterium *Lyngbya* sp., such as oscillatoxin E and 30-methyloscillatoxin D, suppressed Kv1.5 expression in CHO cells with IC50 values of 0.79 ± 0.032 and 1.47 ± 0.138 M, respectively. Researchers exploring innovative approaches for the treatment of atrial tachyarrhythmias should find these findings valuable [52]. Kalkitoxin from *Lyngbya majuscula* was found to be cytotoxic to HCT-116 colon cell lines [124]. Furthermore, with an IC50 value of 5.6 nM, it inhibits hypoxia-induced HIF-1 initiation in T47D breast carcinoma cells [124]. Furthermore, kalkitoxin reduces calcium influx inhibition in primary rat cerebellar granule cell cultures via interacting with voltage-sensitive sodium channels [125]. DA displayed proliferative effects on cancer cell lines such as K562 and EA.hy 927 in vitro [126]. STX have the potential to be used as medicines, such as anesthetics. Pain sensations, muscle spasms, muscle relaxation, and wrinkle reduction may be reduced or completely blocked. STXs possess promising antifungal, antibacterial, antialgal, and antiprotozoal activity in vitro [127]. The E-cadherin–catenin pathway is preferentially impaired by YTXs in epithelial cells, endangering Ecadherin’s tumor-suppressive properties [128]. YTXs have displayed significant cytotoxic effects [129]. In primary cortical neurons, BC3H1 myoblast cells, and glioma cells, YTX produced non-apoptotic cell death [130]. Additionally, it also inhibits the growth of melanoma tumor cells in mouse cells in vivo with minimal damage [131]. YTX appears to impair immunological function by lowering phagocytic activity in the J774 cell line and increasing cytokine expression in J774 phagocyte mammalian cells [132]. Furthermore, reversible T-cell receptor complex downregulation appears to limit the immunological impact on T-lymphocyte EL-4 cells [133]. YTX and its analogs may be used to treat Alzheimer’s disease by lowering levels of t- and β-amyloid, two insoluble forms found in the brain that cause the disease to develop [134]. In addition, YTX may help prevent and treat lipid and glucose metabolism issues in glioma cells, as well as pancreatic and liver transcriptional abnormalities [135]. YTX may also have a minor role as an anti-asthmatic and anti-allergenic drug [136]. Increased muscle contraction, notably in heart tissue, and excessive fluid discharge by gastrointestinal cells have also been shown to have therapeutic effects with CTX [93,94]. On the other hand, this biologically active toxin can be used to investigate the biological function of a variety of human diseases and channelopathies, such as cancer, chronic pain, epilepsy, and cardiac arrhythmias [137,138]. In persons with asthma, BTX improves respiratory irritation symptoms such as cough, nasal irritability, bronchoconstriction, congestion, and/or asthma episodes [139]. As a result, it alters the immune response in alveolar macrophage cells by increasing the production of cytokines (TNF-α and IL-2) involved in immune cell activation, decreasing phagocytosis activity, and playing a key role in pulmonary hypersensitivity inflammation [140,141,142]. In Jurkat E6-1 cells and leukemic T-cell lines, it also has a dose-dependent influence on cell proliferation, causes cell death via apoptosis, and has genotoxic effects [140,143]. BTX-2 has neuro-activation properties and can increase neuronal plasticity, making it potentially beneficial in pharmacological treatments for regaining brain function after a stroke or other traumatic brain injury [144]. A pharmaceutical invention based on BTX derivatives has also been created to treat conditions such as cystic fibrosis and mucociliary dysfunction caused by mucus transport amplification [145]. PLTX reduces cytotoxicity by modulating cytoskeleton distortion and dynamics in intestinal and neuroblastoma cells [87,146]. Furthermore, PLTX, derived from Palythoa clavata polyps and including Symbiodinium dinoflagellate, demonstrated that a pharmaceutical formulation is suitable for use in the treatment of lymphoblastic or myelogenous leukemia [88]. SPX displayed cytotoxic effects [89]. SPX has also been shown to have a neuroprotective impact in Alzheimer’s disease [147].

## 7. Conclusions and Future Prospective

Humans are exposed to phytoplankton toxins through seafood consumption, water intake, and personal contact. Further research employing a cell-based method is required to understand the precise mode of action of marine phytoplankton toxins. The adoption of biological approaches, consisting of nanoparticles that gather toxins, might be an intriguing alternative to toxin reduction. Therefore, this technique should be adopted in the future since it provides a low-cost, efficient, and environmentally friendly way to remove poisons from the environment. To lessen the impact of this toxicity, further research on eliminating these phytotoxins is urgently needed.

Phytoplankton toxins have diverse chemical structures and exhibit various toxic effects. The structure, genesis, symptoms, and molecular mechanisms of tumor promotion activity, as well as ROS toxicity, are discussed. Furthermore, ROS leads to apoptosis via several pathways. Phytoplankton toxins can promote different tumors via different mechanisms. Epigenetics play a crucial role in the development of various malignancies. No studies have been conducted on epigenetic perspectives and tumor development. Therefore, further research is needed to confirm tumor development caused by phytoplankton toxins via epigenetic alterations in mammalian cells.

Despite of their toxicity, phytoplankton toxins are useful in pharmacology because they comprise a diverse spectrum of chemical structures as well as biological features. Phytoplankton have been shown to be a rich source of physiologically active toxins with intriguing biological features that could be exploited in a wide range of therapeutic and medical applications.

## Figures and Tables

**Table 1:** Marine cyanobacterial and diatoms toxins and their toxic target with clinical symptoms.

Toxins	Organisms/Source	Toxic Symptoms	Toxic Target	References
Nodularin	*Nodularia spumigena*		PP inactivation	[17,18,19]
Nodularin	*Nodularia spumigena*	Renal lesions, diarrhea, vomiting, piloerection, weakness, and pallor	Tissue transport and bile anions	[46,47]
Nodularin	*Nodularia spumigena*		Tumor promotion	[18,19]
Oscillatoxin	*Schizothrix calcicola* and *Oscillatoria nigroviridis*	Contact irritants	-	[48]
Lyngbyatoxin-A	*Lyngbya majuscula*	Skin irritant, oral and gastrointestinal inflammation	Tumor promotion	[49]
Lipopolysaccharide	Most of the cyanobacterial species	Allergic, inflammatory, pyrogenic reactions, fever and septic shock syndrome	Tumor promotion	[50]
Aplysiatoxin	*Schizothrix calcicola* and *Oscillatoria nigroviridis*	Inflammation, burning ambiances in the throat and mouth, paraesthesia, abdominal pain, vomiting, diarrhea, convulsions, and low blood pressure	Tumor promotion	[51]
Aplysiatoxin	*Schizothrix calcicola* and *Oscillatoria nigroviridis*	Gastrointestinal symptoms, including diarrhea, nausea, and vomiting	blocked potassium channel Kv1.5	[52]
Aplysiatoxin	*Schizothrix calcicola* and *Oscillatoria nigroviridis*		Sodium channel blocked	[51]
Kalkitoxin	*Lyngbya majuscula* and *Trichodesmium* spp.	Neurotoxic	Sodium channel blocked	[53]
Antillatoxin	*Lyngbya majuscula*	Ichthyotoxicity and neurotoxicity	Sodium channel blocked, Neurotoxicity	[54]
Domoic acid	*Pseudo-nitzschia australis*, *Pseudo-nitzschia calliantha*, *Pseudo-nitzschia cuspidate*, *Pseudo-nitzschia delicatissima*, *Pseudo-nitzschia fraudulenta*, *Pseudo-nitzschia galaxiae*, *Pseudo-nitzschia multiseries*, *Pseudo-nitzschia multistriata*, *Pseudo-nitzschia pseudodelicatissima*, *Pseudo-nitzschia pungens*, *Pseudo-nitzschia seriata*, and *Pseudo-nitzschia turgidula*	Abdominal pains, vomiting, and diarrhea, severe headaches, confusion, agitation, somnolence (sleepiness), memory loss, coma, Ataxia (incoordination), excessive scratching, sleepiness, tremors, heart, Seizures, spells of significant lethargy and inappetence, central blindness, vomiting, blepharospasm, muscular twitching, and aberrant behavior difficulties, convulsions, and mortality	Sodium channel blocked and Glutamate receptors	[55]
Saxitoxins	*Lyngbya wollei*, *Cylindrospermopsis raciborskii*, *Anabaena circinalis*, and *Aphanizomenon flos-aquae*	Respiratory arrest, cardiovascular shock, tickling sensations in the mouth, lips, and tongue, numbness in the extremities, breathing difficulties, gastrointestinal problems, and full paralysis	Sodium channel blocked, Voltage-dependent sodium channel Site 1	[56,57]

**Table 2:** Marine dinoflagellates toxins and their toxic target with clinical symptoms.

Toxins	Organisms/Source	Toxic Symptoms	Toxic Target	References
Okadaic acid	*Dinophysis* sp. and *Prorocentrum lima*	Incapacitating diarrhea, nausea, vomiting, and abdominal pain	PP inactivation, Oxidative damage, cellular dysfunction, cell cycle, gene expression, inhibit DNA repair mechanism	[76,77,78]
Dinophysistoxins Okadaic acids	*Dinophysis* spp. *Prorocentrum* spp.	Gastrointestinal illness, nausea, vomiting, and abdominal pain	Ser/thr protein phosphatases	[79,80]
Azaspiracid	*Protoperidinium crassipes*	Severe diarrhea, vomiting, nausea, stomach cramps, and neurotoxicity	Tumor promotion	[81]
Ciguatoxin	*Gambierdiscus toxicus*	Neurological, gastrointestinal, and cardiovascular problems	Sodium channel blocked, Voltage-dependent sodium channel Site 5	[82,83,84]
Saxitoxins	*Alexandrium* spp. *Gymnodinium* spp. *Pyrodinium* spp.	Respiratory arrest, cardiovascular shock, tickling sensations in the mouth, lips, and tongue, numbness in the extremities, breathing difficulties, gastrointestinal problems, and full paralysis	Sodium channel blocked, Voltage-dependent sodium channel Site 1	[56,57]
Brevetoxin	*Karenia brevis* *Gymnodinium breve*	Slighter gastroenteritis with neurologic indicators, Nausea, tingling and numbness in the perioral area, loss of motor function, and acute muscular pain	Sodium channel blocked, Voltage-dependent sodium channel Site 5	[85]
Yessotoxins	*Gonyaulax spinifera*, *Lingulodinium polyedrum*, and *Protoceratium reticulatum*	Motor discoordination	Sodium channel blocked	[86]
Palytoxin	*Ostreopsis mascarenensis*, *O. siamensis*, *O. lenticularis*, *O. fattorussoi*, and *O. ovata*,	Fever, ataxia, inactivity, drowsiness, and limb weakness	Sodium channel blocked	[87,88]
Spirolides	*Alexandrium ostenfeldii*, *Alexandrium peruvianum*, and *Karenia selliformis*	Neuron and astrocytes damage	Sodium channel blocked	[89]

## Data Availability

Not applicable.

## Abbreviations

ATM: ataxia telangiectasia mutated; AZA, azaspiracid; CAT, catalase; CTX, Ciguatoxin; DA, Domoic acid; IL2, interleukin-2; JNK, c-Jun N-terminal kinase; mitogen activated protein kinase; MMP, mitochondrial membrane potential; OA, okadaic acid; PTX, palytoxin; ROS, reactive oxygen species; SOD, superoxide dismutase; SPX, Spirolides; STX, Saxitoxins; TNF-α, tumor necrosis factor-α; YTXs, yessotoxins.
